# The Reliability of a New Device for Measuring the Maximum Bite Force

**DOI:** 10.1155/2022/3272958

**Published:** 2022-01-13

**Authors:** Santosh R. Patil, G. Maragathavalli, D. N. S. V. Ramesh, Giridhar S. Naidu, Mohammad Khursheed Alam, Ibrahim A. AlZoubi

**Affiliations:** ^1^Department of Oral Medicine and Radiology, Saveetha Dental College and Hospitals, Chennai, India; ^2^Department of Oral Medicine and Radiology, A.M.E's Dental College and Hospital, Raichur, India; ^3^Department of Oral Medicine and Radiology, New Horizon Dental College and Research Institute, Chhattisgarh, India; ^4^Department of Preventive Dentistry, College of Dentistry, Jouf University, Saudi Arabia

## Abstract

**Objective:**

To test the inter- and intraexaminer reliability of a recently developed instrument for measuring the maximum bite force (MBF). *Material and Methods*. Sixty patients who were clinically confirmed as having Oral Submucous Fibrosis (OSMF) and 60 healthy controls were included in this study. For each subject, age, gender, weight, height, and body mass index (BMI) were recorded. The maximum bite force was recorded in alternate order with a bite force sensor (D1) and an occlusal force meter (D2). Bite force was measured in the first molar region. Pearson's correlation coefficient and kappa statistic were applied to assess the reliability between D1 and D2 in the assessment of maximum bite force. The independent *t*-test was performed to find the statistical significance between the two study groups. The paired *t*-test was applied to find out the difference between the right and left disease in groups of two devices separately. The one-way analysis of covariance (ANOVA) was performed to find the significant difference between grades of OSMF.

**Results:**

The results of the kappa values were 0.8531 ± 0.0724 and 0.7336 ± 0.0737 for interdevice reliability in OSMF patients in right and left sides. Similar findings were obtained in right and left sides of healthy individuals (0.7549 ± 0.0816 and 0.9440 ± 0.0806) and in the total sample (0.8132 ± 0.0544 and 0.8303 ± 0.0538). Pearson's correlation coefficient between two devices revealed a high and significant positive correlation between D1 and D2 separately and in the whole sample.

**Conclusion:**

The observations of the present study suggest that the bite force sensor can be used as a reliable device for measuring bite force.

## 1. Introduction

Bite force is one of the indices of the functional state of the masticatory complex resulting from the activity of jaw muscles. Bite force may be considered a significant factor in assessing the disruption of the stomatognathic system and evaluation of particular bite force level antiquated extensively in dental practice, to know the mechanics of mastication [1].

Data in the literature accentuates significant factors that influence bite force measurements, like age, sex, body mass index, craniofacial morphology, occlusion, periodontal status of an individual, temporomandibular disorders and pain, and dentition status [2].

The enormous difference in bite force values banks on various circumstances pertinent to the anatomical and physiologic attributes of the subjects. Ancillary to these biological components, mechanical elements composed of various recording devices, location of recording devices in the maxillary or mandibular arch, unilateral or bilateral measurements with the aid of acrylic splints, and wide opening of the mouth were emphasized as factors influencing the bite force [3]. Hence, various researchers noted a varying spectrum of maximal bite force values in different studies [1, 4].

A number of tools with various designs and working principles have been used to assess the bite force values in human beings. The bite force analysis is usually carried out directly using a transducer which is placed between teeth in either anterior or posterior segment of the jaws. This approach of force appraisal seems to be a conducive method of evaluating the submaximal force. A substitutive approach is indirect assessment of the bite force by advocating other physiologic variables known to be functionally pertinent to the force generation [5].

The interest regarding the bite force has been widely discussed in the literature since very long time from the past. In the associated studies, a wide variety of techniques and equipment for the estimation of bite forces have been mentioned. These devices differ from a simple spring to a wide variety of complex electronic appliances [5].

Several devices have been used to measure bite forces directly including biting fork, strain gauge transducers, quartz and foil transducers, pressurized rubber tube, gnathodynamometer, pressure-sensitive sheet, and force sensing resistors [5].

Even though a variety of devices are available to assess the bite force, no single tool is capable of recording all the required forces and it could be difficult to choose a recording device which fulfils the objective of recording bite force. In the present study, we evaluated the reliability of a recently developed instrument for measuring the maximum bite force. The bite force sensor used in this study is an electromechanical device which measures the mechanical deflections in the jaws by analog output which comes in millivolts. Analog output is converted into digital mathematical values in the display unit. The force transducer occlusal force meter is a very popular tool used for the evaluation of bite force. The accuracy and repeatability of this occlusal force gauge have been previously confirmed [6–13]. The kappa values of this study showed perfect agreement between two devices, and the correlation coefficient between two devices revealed a high and significant positive correlation between both devices.

## 2. Material and Methods

In the present study, 60 patients who were clinically confirmed as having OSMF and 60 healthy controls were included in this study. The OSMF patients were graded according to the previous classification. The patients were divided into four groups on the basis of interincisal mouth opening, i.e., group I (mouth opening, 35 mm), group II (mouth opening between 30 and 35 mm), group III (mouth opening between 20 and 30 mm), and group IV (mouth opening, 20 mm) [14]. The inclusion criteria considered in the selection of the participant are as follows: angle class I molar relationship without an anterior or posterior crossbite or open bite; class 1 facial profile and normal facial height and no history of orthodontic therapy; no missing teeth in the molar region; no pain related to the molars; no heavily restored teeth in the molar region; no gingival inflammation, no periodontal pathology, and absence of mobility of the teeth; and no reported systemic disease (chronic arthritis) or apparent facial asymmetry that could influence the registration of bite force. Subjects with parafunctional habits, pathological wearing facets, or any other soft tissue pathologies; temporomandibular joint dysfunction; and systemic disease that may influence the neuromuscular system (such as Parkinson's disease) were excluded from this study [15]. For each participant, age, sex, body weight, height, and body mass index (BMI) were recorded.

**Figure 1:**
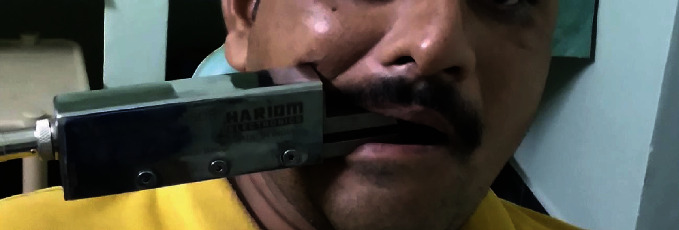
Bite force sensor (D1).

**Figure 2:**
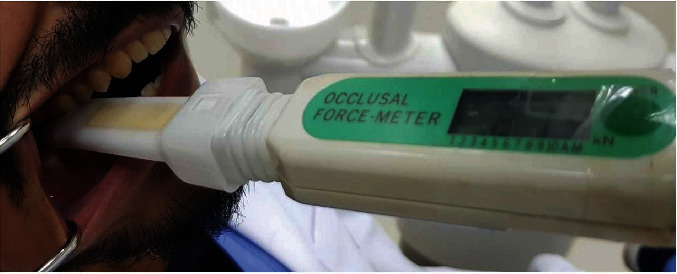
Occlusal force meter (D2).

The devices were placed in the site of the first molar and in the site of incisors for recording posterior for anterior bite force, respectively. The participant was made to sit in upright position, with Frankfort's plane nearly parallel to the ground without any head support. The participants were instructed to bite as heavy as they could on the recording surface of the device. The maximum bite force value of the three tests, with 45-second rest between each recording, was documented to be the maximum bite force for right and left sides. The maximum bite force was recorded in alternate order with a bite force sensor (D1) (Figure 1) and an occlusal force meter (D2) (Figure 2).

Measurements were repeated after a 14-day interval in 20 randomly selected patients to confirm the reliability and tested using the intraclass correlation coefficient.

### 2.1. Statistical Analysis

Pearson's correlation coefficient and kappa statistic were applied to assess the reliability between D1 and D2 in the assessment of maximum bite force. The independent *t*-test was performed to find the statistical significance between two study groups. The paired *t*-test was applied to find out the difference between the right and left disease in groups of two devices separately. The one-way analysis of covariation was performed to find the significance difference between grades of OSMF by statistical software, i.e., SPSS 20.00 version. The statistical significance was set at 5% level of significance (*p* < 0.05).

## 3. Results

The results of the kappa values were 0.8531 ± 0.0724 and 0.7336 ± 0.0737 for interdevice reliability in OSMF patients on right and left sides. Similar findings were obtained in right and left sides of healthy individuals (0.7549 ± 0.0816 and 0.9440 ± 0.0806) and in the whole sample (0.8132 ± 0.0544 and 0.8303 ± 0.0538). The intraclass correlation coefficient ranged from 0.89 to 0.96. No significant difference was observed between OMFS patients and healthy individuals with mean age (*t* = −0.7973, *p* = 0.4269), mean weight (kg) (*t* = −0.1661, *p* = 0.8684), mean height (m) (*t* = 1.9270, *p* = 0.0564), mean BMI (*t* = −1.2617, *p* = 0.2096), and mean number of intact teeth (*t* = 0.1506, *p* = 0.8805) at 5% level of significance (Table 1).

Correlation coefficients between physical characteristics (age, weight, height, and BMI) and average MBF are shown in Table 2. There was a positive correlation between average MBF and all physical characteristics, but it was not significant. On applying the independent *t*-test, no significant difference was observed between OSMF and healthy individuals with respect to mean MBF obtained by D2 (*t* = 1.6690, *p* = 0.0978) on the right side and mean MBF (*t* = 1.1887, *p* = 0.2369) on the left side at 5% level of significance. Similarly, no significant difference was observed between OSMF and health subjects with respect to mean MBF obtained by D1 (*t* = 1.6876, *p* = 0.0941) on the right side and mean MBF (*t* = 1.6598, *p* = 0.0996) on the left side at 5% level of significance (Table 3).

On applying the dependent *t*-test, no significant difference was observed between right and left sides with respect to mean MBF obtained by the standard device (*t* = 1.2058, *p* = 0.2327) in OSMF patients and mean MBF (*t* = 0.2642, *p* = 0.7925) in healthy individuals at 5% level of significance. Similarly, no significant difference was observed between right and left sides with respect to mean MBF obtained by D1 (*t* = −2.0000, *p* = 0.0501) in OSMF patients and mean MBF (*t* = −1.2253, *p* = 0.2253) in healthy individuals at 5% level of significance (Table 4).

On comparing different grades of OSMF with all variables, a nonsignificant difference between OSMF grades with age, BMI, number of intact teeth, and MBF scores on right and left sides by both devices (i.e., D1 and D2) at 5% level (*p* > 0.05) was noted (Table 5).

## 4. Discussion

Several limitations have been reported for the recording of a maximum voluntary clenching such as the possibility of dental fractures on the metal surfaces of the transducer, pain, discomfort, fear preventing a maximal performance, and technical limitations of the instrument [16, 17].

Borelli carried out the first experimental study defining the intraoral forces, which was performed by those who fabricated a gnathodynamometer. He fixed various weights to a cord, which crossed over the molar teeth of the open lower jaw and with closing of the jaw. The first scientific evaluation of forces was carried out by Black. He reached to his results by fabricating a new type of gnathodynamometer. Various scientists later extended investigation regarding this topic and fabricated the lever-spring, manometer-spring and lever, and micrometered appliances. In today's practice, sensitive electronic devices are commonly used which are both authentic and precise for the analysis of routine bite force [17].

A biting fork is an aluminum device, with flat, cantilevered surfaces with cemented strain gauges that record the deformation produced during biting. It produces 4 mm separation between teeth. Quartz or foil transducers operate on the piezoelectric principle. They produce a signal in the form of a small electrical charge and, therefore, must be used with a charge amplifier. If a steady load is applied to a piezoelectric material, a charge will appear when the load is applied but then will fade away. When the load is released, an equivalent charge spike is observed in the opposite direction. The output from this transducer consisted of a summation of laterally and axially directed occlusal forces [5, 17].

A pressure-sensitive sheet is based on a material used industrially and known as Prescale. When the sheet is bitten, microcapsules are broken to release staining granules. The occlusal contacts can be detected by a color-developing chemical reaction. The occlusal contact area and pressure, from which the bite force is calculated, are evaluated with an occlusion pressure graph based on the degree of coloring. The film is unaffected by intraoral humidity and temperature change, and the velocity and duration of the force applied to it have a negligible influence on color formation [17].

Another pressure-sensitive device used for bite force measurement is a fiber-reinforced pressurized rubber tube connected to a pressure sensing element. Changes in pressure are transformed into electrical signals and transferred to a digital strain indicator [18].

Recently, a bite force sensor based on force sensing resistors was used clinically. Force sensing resistors are a polymer thick film (PTF) device which exhibits a decrease in resistance with an increase in the force applied to the active surface [19].

Instead of measuring bite forces directly, electromyographic (EMG) activity of the surface elevator muscles of the mandible has been used as an indirect estimator of bite forces. EMG potentials can be directly picked up from the cutaneous projection of the muscular belly in a noninvasive and in a safer way [20].

In the present study, we compared the reliability of a new bite force sensor (Hariom Electronics, Vadodara, Gujarat, India) in comparison with a force transducer occlusal force meter (GM10, Nagano Keiki Co. Ltd., Tokyo, Japan).

The body of the bite force sensor device used in this study is made of stainless steel, and the sensing area of jaws is made of heat treated and annealed special graded alloy tool steel. It has 5 pin connectors for transferring signals and excitation voltage from and to the process indicator unit. This device was already used to measure bite force in some of the previous studies [12, 21, 22].

## 5. Conclusion

The observations of the present study suggest that the bite force sensor can be used as a reliable device for measuring bite force. Similar studies in a larger and different population to evaluate bite force with this new device needs to be carried out to further check its efficacy and reliability.

## Figures and Tables

**Table 1:** Distribution of subjects according to mean height, weight, BMI, and the number of intact teeth present in OSMF and healthy groups.

Variables	OSMF group		Healthy group		*t* value	*p* value
	Mean	Std. dev.	Mean	Std. dev.		
Age	38.32	5.40	39.07	4.89	-0.7973	0.4269
Weight (kg)	69.40	6.30	69.60	6.88	-0.1661	0.8684
Height (m)	1.72	0.09	1.69	0.07	1.9270	0.0564
BMI	23.72	3.57	24.51	3.21	-1.2617	0.2096
Intact teeth, number	29.65	2.34	29.60	1.06	0.1506	0.8805

Std. dev: standard deviation.

**Table 2:** Correlation coefficient between bite force and physical characteristics.

	*R* ^2^	*p* value
Weight	0.151	0.329
Height	0.264	0.198
BMI	0.024	0.877

**Table 3:** Comparison of maximum bite force between the OSMF group and healthy groups on the right and left sides in D2 and D1 by the independent *t*-test.

Device	Sides	OSMF group		Healthy group		*t* value	*p* value
		Mean	Std. dev.	Mean	Std. dev.		
D2	Right side	629.12	35.67	619.84	24.17	1.6690	0.0978
	Left side	624.72	27.09	619.34	22.31	1.1887	0.2369
D1	Right side	625.84	27.44	617.52	26.55	1.6876	0.0941
	Left side	631.61	26.59	622.00	36.13	1.6598	0.0996

Std. dev: standard deviation.

**Table 4:** Comparison of maximum bite force between the right and left sides in the OSMF group and healthy groups in D2 and D1 by the dependent *t*-test.

Device	Groups	Right side		Left side		*t* value	*p* value
		Mean	Std. dev.	Mean	Std. dev.		
D2	OSMF group	629.12	35.67	624.72	27.09	1.2058	0.2327
	Healthy group	619.84	24.17	619.34	22.31	0.2642	0.7925
D1	OSMF group	625.84	27.44	631.61	26.59	-2.0000	0.0501
	Healthy group	617.52	26.55	622.00	36.13	-1.2253	0.2253

Std. dev: standard deviation.

**Table 5:** Comparison of grades of OSMF with all variables by the one-way ANOVA test.

Variables	Summery	Grade 0	Grade 1	Grade 2	Grade 3	*F* value	*p* value
Age	Mean	38.63	38.09	38.15	38.32	0.0637	0.9383
	SD	5.40	5.91	4.83	5.40		
Weight (kg)	Mean	68.92	69.78	69.62	69.40	0.1170	0.8898
	SD	6.40	7.01	5.09	6.30		
Height (m)	Mean	1.74	1.70	1.73	1.72	1.3894	0.2575
	SD	0.10	0.08	0.09	0.09		
BMI	Mean	23.10	24.45	23.61	23.72	0.8463	0.4343
	SD	3.77	3.44	3.43	3.57		
Intact teeth, number	Mean	30.38	29.13	29.23	29.65	1.9885	0.1463
	SD	3.09	1.18	2.09	2.34		
Right side-D2	Mean	617.33	637.39	636.27	629.12	2.2838	0.1111
	SD	25.77	27.96	55.56	35.67		
Left side-D2	Mean	614.78	633.90	626.85	624.72	3.1053	0.0522
	SD	26.44	25.09	27.40	27.09		
Right side-D1	Mean	617.13	634.17	627.18	625.84	2.3939	0.1004
	SD	26.38	25.93	29.12	27.44		
Left side-D1	Mean	623.62	639.49	632.42	631.61	2.1837	0.1220
	SD	17.77	32.61	25.96	26.59		

Std. dev: standard deviation.

## Data Availability

All data are available within the manuscript.
